# Influences of conservation measures on runoff and sediment yield in different intra-event-based flood regimes in the Chabagou watershed

**DOI:** 10.1038/s41598-021-95111-6

**Published:** 2021-08-02

**Authors:** Shan-Shan Wang, Zhan-Bin Li, Le-Tao Zhang, Bo Ma

**Affiliations:** 1 State Key Laboratory of Soil Erosion and Dryland Farming on the Loess Plateau, Institute of Soil and Water Conservation , Chinese Academy of Sciences and Ministry of Water Resources, Xinong Rd. 26, Yangling, 712100 Shaanxi China; 2 University of Chinese Academy of Sciences, Beijing, 100049 China; 3 College of Environment and Planning, Henan University, Kaifeng, 475004 Henan China

**Keywords:** Ecology, Hydrology

## Abstract

The Loess Plateau in China has suffered severe soil erosion. To control soil erosion, extensive conservation measures aimed at redistributing rainfall, hindering flow velocity and intercepting sediment were implemented on the Loess Plateau. To accurately evaluate the combined effect of conservation measures in the Chabagou watershed, this study classified intra-event-based floods into four regimes via cluster and discriminant analyses. Regime A was characterized by short flood duration and low erosive energy, regime B was characterized by short flood duration and high erosive energy, regime C was characterized by long flood duration and low erosive energy, and regime D was characterized by long flood duration and high erosive energy. The results indicated that peak discharge (*q*_*p*_), runoff depth (*H*), mean discharge (*q*_*m*_), and runoff erosion power (*E*) decreased by 75.2%, 56.0%, 68.0% and 89.2%, respectively, in response to conservation measures. Moreover, area-specific sediment yield (*SSY*), average suspended sediment concentration (*SCE*), and maximum suspended sediment concentration (*MSCE*) decreased by 69.2%, 33.3% and 11.9%, respectively, due to conservation measures. The nonlinear regression analysis revealed a power function relationship between *SSY* and *E* in both the baseline (1961–1969) and measurement period (1971–1990) in all regimes. Conservation measures reduced sediment yield by not only reducing the runoff amount and soil erosion energy but also transforming the flood regime, for example, transforming a high-sediment-yield regime into a low-sediment-yield regime. Moreover, conservation measures altered the *SSY*-*E* relationship in regime A, whereas no obvious difference in regime B or C/D was observed between the measurement period and the baseline period. This study provides a better understanding of the mechanism of runoff regulation and the sediment yield reduction under comprehensive conservation measures in a small watershed on the Chinese Loess Plateau.

## Introduction

Soil erosion, which includes the processes of soil destruction, peeling, transport and deposition by external forces, is extremely complicated^1–3^. Exploring the characteristics of runoff and soil erosion processes is imperative for a better understanding of the mechanisms of soil erosion. In recent decades, numerous studies have focused on the spatial and temporal heterogeneity of rainfall and other hydrological processes in watersheds^4–8^, and the results have improved the accuracy of hydrological models in simulating the processes of rainfall and runoff generation^9–12^. In addition, an increasing number of studies have focused on the effects of hydrological regimes on soil erosion and sediment behavior^13,14^. Surface runoff is an erosive agent and medium for water erosion, and its flow determines the capacity for erosion and sediment transport^15^, parameters of which are widely used as indicators in research on sediment flow behavior^13,15^. However, the relationships between soil erosion and hydrological processes have not been thoroughly examined, with studies and data remaining limited^13,15^.

The Loess Plateau in China, which is highly fragmented by gullies, has suffered severe soil erosion. Since the 1970s, a series of conservation measures, including terracing, afforestation, and damming, have been implemented on the Loess Plateau to prevent soil and water loss and maintain agricultural productivity^16,17^. By applying the WATEM/SEDEM erosion model, Boix-Fayos et al.^18^ found that changes in land use in the absence of check dams decreased the sediment yield by 54%, whereas with check dams but without land use changes, 77% of the sediment yield was retained. Terraces, a type of conservation measure, can favor water infiltration, soften steep mountainous slopes and reduce runoff and soil erosion^19^. Mulching also has profound influences on infiltration, surface runoff, soil moisture and erosion; mulch cover of 2 t/ha and 4 t/ha has been found to reduce the runoff peak by 21% and 51%, respectively^20^. Conservation measures can not only reduce soil erosion but also modify flow regimes^21–23^. However, research is lacking on the effect of conservation measures on the relationship between runoff and sediment in different surface runoff regimes. Given the complexity of conservation measures on the Loess Plateau, it is difficult to isolate the influences of individual measures on stream flow^23^. Therefore, it may be beneficial to investigate the combined effects of conservation measures on the sediment flow of the watershed and the runoff-sediment relationship by analyzing data from a watershed outlet station.

The aims of this study were to (1) assess the integrated effects of conservation measures on surface runoff and sediment in the Chabagou watershed, (2) classify intra-event-based floods and explore the influence of conservation measures on the intra-event-based flood regime, and (3) evaluate the changes in the runoff-sediment relationship due to conservation measures in different flood regimes. This study is expected to provide a better understanding of the mechanism of sediment reduction due to conservation measures at the watershed scale.

## Study area, data source and treatments

### Study area

**Figure 1:**
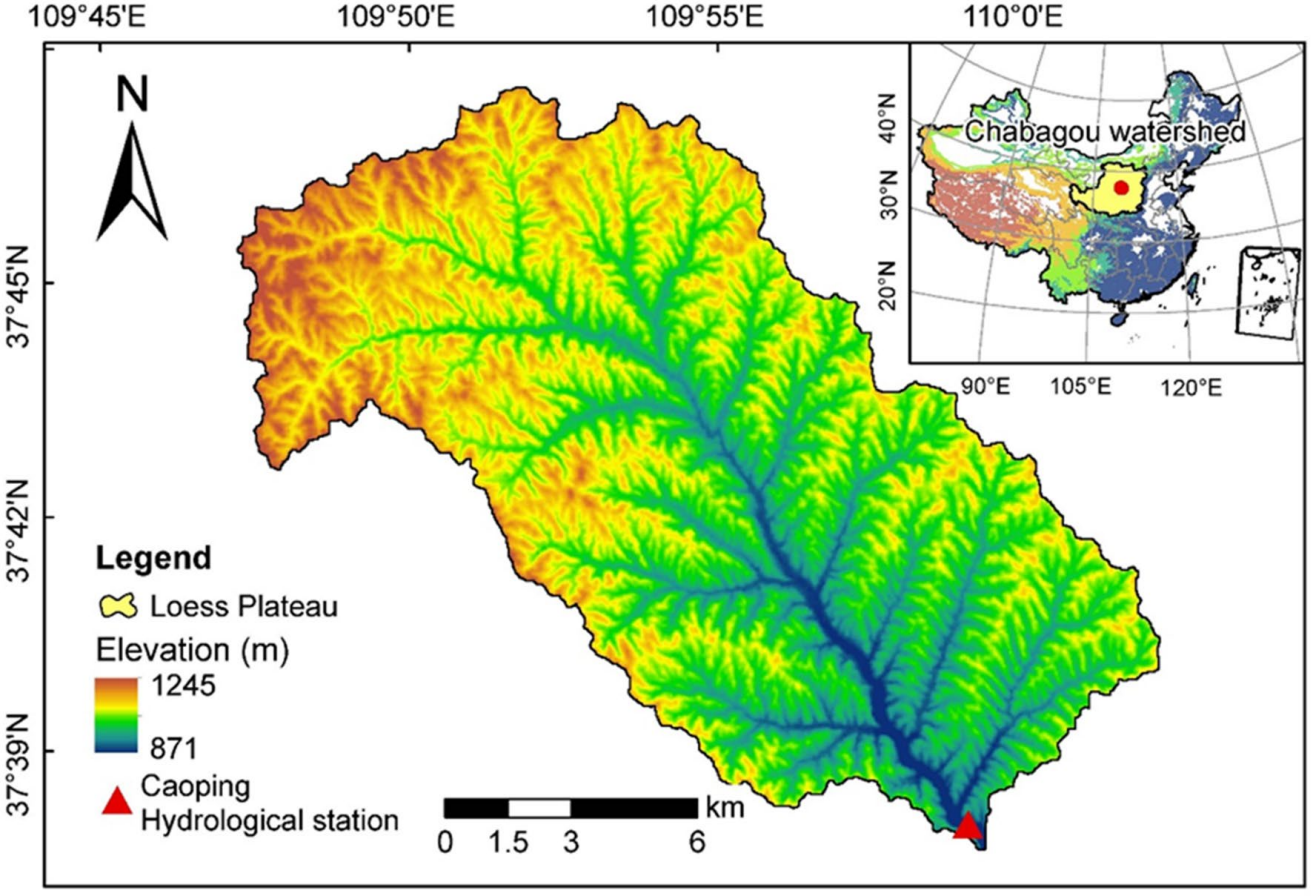
Map of the Chabagou watershed. The map was generated with ESRI ArcMap 10.5 software (http://www.esri.com/arcgis/) with terrain data acquired from the Advanced Land Observing Satellite (ALOS) Phased Array type L-band SAR (PALSAR) Radiometric Terrain Corrected high-resolution dataset^42^.

The Chabagou watershed has nested hydrological stations, long time series of measured hydrological and sediment data and complete meteorological data, which are important in geomorphic process research, hydrological simulation and sediment research in the hilly and gully region of the Loess Plateau. The Chabagou watershed (109°47′ E, 37°31′ N), which is part of the first region of the gullied and rolling Loess Plateau, is a first-order tributary region of the Dali River. The Chabagou watershed, with an area of 205 km^2^ and a channel length of 26.5 km, is symmetric in shape, and its elevation ranges from 900 to 1100 m. Its average annual precipitation is approximately 450 mm. The rainfall distribution is uneven throughout the year, with 70% of the total rainfall being concentrated from June to September, mostly as strong intensity and short-duration rainstorms. The temperature varies from − 27 to 38 °C, and the annual average temperature is 8 °C. Due to loose soil, sparse vegetation, heavy rainfall intensity, etc., the region suffers severe soil erosion. The average annual erosion modulus is 22,200 t km^−2^, and the maximum and minimum annual erosion moduli are 71,100 t km^−2^ and 2110 t km^−2^, respectively. The Caoping hydrological station (Fig. 1), which services a catchment area of 187 km^2^, a channel length of 24.1 km and an average gully channel gradient of 7.57‰, is set at the watershed outlet to observe the hydrology and sediment conditions.

During the 1961–1969 observation period, the Chabagou watershed was in a near-natural state, with little artificial disturbance^24^. A series of conservation measures were initiated in the Chabagou watershed in 1970. By 1990, there were 21.13 km^2^ of terraced fields, 8.63 km^2^ of afforestation, 2.13 km^2^ of planted grass and 4.07 km^2^ of dammed land in the Chabagou watershed. The total treatment area was 35.96 km^2^, which accounted for 17.54% of the watershed. Based on the double-accumulative curve method and considering that engineering measures such as dams and terraces reached their peak in the 1970s, Qi Junyu concluded that soil and water conservation measures were effective in 1970^25^. Hence, 1961–1969 and 1971–1990 were regarded as the baseline period and measurement period, respectively^25^.

### Data source and treatments

The hydrological and sediment data are from a hydrological experiment at the Zizhou runoff experimental station, which was set up by the Yellow River Water Conservancy Committee (1961–1990, excluding 1970) (Loess Plateau Data Center, National Earth System Science Data Sharing Infrastructure, National Science & Technology Infrastructure of China (http://loess.geodata.cn)). The collection of all the water and sediment data, including water level, flow rate, sediment concentration and sediment yield, as well as sampling and experimental analysis, was performed in strict accordance with national standards^26^.

According to relevant test standards of the hydrological station^27^, a flood runoff event with a runoff depth ≥ 0.1 mm, a peak flow rate ≥ 1 m^3^/s, and a duration ≥ 450 min was defined as a main channel flood event (at Caoping hydrological station). A total of 49 flood events from 1961 to 1969 and 82 flood events from 1971 to 1990 were selected.

Indicators such as flood duration (*T*, min), time-to-peak (*T*_*p*_, min), duration of recession (*T*_*r*_, min), peak discharge (*q*_*p*_, m^3^ s^−1^), runoff depth (*H*, mm), mean discharge (*q*_*m*_, m^3^ s^−1^), flow variability (*FV*), and runoff erosion power (*E*, m^4^ s^−1^) were selected to reflect the runoff characteristics of intra-event-based floods. *E* is the product of the peak discharge and runoff depth, and it represents the average efficiency of the combined effects of natural rainfall characteristics and the underlying surface characteristics of the basin on erosion and sediment yield in the basin^28^.

The sediment characteristics of intra-event-based floods were reflected by indicators such as area-specific sediment yield (*SSY*, t km^−2^), average suspended sediment concentration (*SCE*, kg m^−3^), maximum suspended sediment concentration (*MSCE*, kg m^−3^) and sediment variability (*SCV*). The calculation formulas of these indicators were as follows:

$$\mathrm{Mean} \, \mathrm{runoff} \, \mathrm{ depth}: H=\frac{\sum q\Delta t}{A}$$

$$\mathrm{Mean} \, \mathrm{ discharge}:{q}_{m}=\frac{\sum q\Delta t}{T}$$

$$\mathrm{Flow } \, \mathrm{variability}:FV=\frac{{q}_{p}}{{q}_{m}}$$

$$\mathrm{Runoff} \, \mathrm{ erosion } \, \mathrm{power}: E={q}_{p}H$$

$$\mathrm{Sediment} \, \mathrm{ yield}: ESY=\sum Sq\Delta t$$

$$\mathrm{Area}-\mathrm{specific} \, \mathrm{ sediment} \, \mathrm{yield}: SSY=\frac{ESY}{A}$$

$$\mathrm{Average } \, \mathrm{suspended } \, \mathrm{sediment } \, \mathrm{concentration}: SCE=\frac{SSY}{H}$$

$$\mathrm{Sediment } \, \mathrm{variability}: SCV=\frac{SCE}{MSCE}$$

where *Δt* represents the time interval of hydrological observation (min); *q*_*p*_, *q*_*m*_, *H*, *FV* and *E* represent the peak discharge (m^3^ s^−1^), mean discharge (m^3^ s^−1^), runoff depth (mm), flow variability and runoff erosion power (m^4^ s^−1^), respectively; and *S*, *SCE*, *MSCE*, *ESY*, *SSY* and *SCV* represent the instantaneous sediment concentration (kg m^−3^), average suspended sediment concentration (kg m^−3^), maximum suspended sediment concentration (kg m^−3^), sediment yield (t), area-specific sediment yield (t km^−2^) and sediment variability, respectively.

### Study method

Based on the flood runoff characteristics, intra-event-based flood events in 1961–1969 were classified into different flood process regimes using cluster analysis and discriminant analysis^29,30^. Zhang^13,31^ and other researchers^32,33^ performed similar research on the classification of flood events using cluster analysis, and they adopted flood duration, runoff depth and peak discharge as indices. Runoff depth can reflect the precipitation amount and the influence of the underlying surface of a watershed on the rainfall redistribution, and peak discharge can reflect the temporal and spatial distributions of rainfall and the effect of the underlying surface of the watershed on the confluence process^28^. Flood duration is one of the main indices of rainfall type^34^. Thus, we adopted the following variables as classification indices: flood duration (*T*), runoff depth (*H*) and peak discharge (*q*_*p*_). After repeated trial and error tests, hierarchical cluster analysis and discriminant analysis were selected to classify the flood events at the Caoping hydrological station in 1961–1969.

The basic idea of a hierarchical cluster analysis is to first cluster variables with similar distances according to distance and then sequentially cluster variables with more distant distances until each variable is placed into a suitable cluster. The process of hierarchical cluster analysis in SPSS is as follows: Assuming that there are n variables in a data set, the first step is to determine the basic meaning of the distance and the calculation method of the distance between classes. In the second step, these n variables are grouped into a class, and there are n classes in total. In the third step, variables with similar distances are grouped into one class according to the calculated interclass distance, and other variables are still classified into one class. In this case, there are n-1 classes. The fourth step is to further aggregate the classes that are near each other, yielding n-2 classes. The process continues sequentially until all of the data are fully grouped into a category. The Ward method and Euclidean distance were used in the hierarchical cluster analysis, and the Fisher discriminant function was used in the discriminant analysis. Based on this classification, the intra-event-based flood events in 1971–1990 were discriminated.

Cluster analysis, discriminant analysis, regression analysis and other data analysis processes were performed using SPSS 18.0. Origin 12.5 was used to prepare figures.

## Results

### Effects on intra-event-based flood runoff and sediment characteristics

Between the 1960s and 1990s, there was no significant change in rainfall in the Chabagou watershed^35^. The mean values of runoff and sediment transport in the baseline period and measurement period were calculated. Regardless of rainfall influence, the effect of conservation measures was assessed by the time series contrasting method^25^.

Table 1 shows the statistics of the characteristics of event-based flood flows and sediment in 1961–1990 (excluding 1970). Compared with those in the baseline period, *T* and *T*_*r*_ in the measurement period increased by 16.54% and 29.21%, respectively; however, *T*_*p*_ decreased by 55.52% in the measurement period, which showed that the soil and water conservation measures extended the flood duration while reducing the time of increased discharge. Under identical rainfall conditions, long-duration runoff with less time for increased discharge could cause less erosion than short-duration runoff with more time for increased discharge^36^. Hence, the conservation measures reduced soil erosion by prolonging the flood duration and reducing the time to peak. In addition, the hydrodynamic indices *q*_*p*_, *H* and *q*_*m*_ were 75.2%, 56.0% and 68.0% lower, respectively, in the measurement period than in the baseline period. Moreover, *E* in the measurement period was only 10.2% that in the baseline period. The results showed that the conservation measures greatly reduced the hydrodynamic energy and thus soil erosion. In addition, the relative erosion indicators *SSY*, *SCE* and *MSCE*, decreased 69.2%, 33.3%, and 11.9%, respectively, in the measurement period compared with the baseline period, which indicated that the conservation measures significantly reduced soil erosion and decreased the mean sediment concentration, although the reduction in the maximum sediment concentration was relatively small. The conservation measures, especially the engineering measures, reduced the runoff velocity, extended the flood duration, and reduced the peak discharge, which sharply reduced the runoff erosion power^37,38^. As a consequence of the decrease in erosive energy, soil erosion was diminished.

**Table 1:** Descriptive statistics of the characteristics of event-based flood flows and sediment in 1961–1990 (excluding 1970).

Statistic	*T*	*T*_*p*_	*T*_*r*_	*q*_*p*_	*H*	*q*_*m*_	*FV*	*E*	*SSY*	*SCE*	*MSCE*	*SCV*
**1961–1969**												
Minimum	465	3	399	1.19	0.14	0.50	2.2	0.0002	12.1	58.0	98.5	1.1
Maximum	3360	1182	2865	1520	36.26	48.08	46.5	43.1	28,143.8	976.4	1220.0	2.3
Mean	1540.1	230.0	1310.0	209.51	4.97	11.09	17.7	2.7	3640.0	649.6	825.1	1.3
Std. deviation	703.4	266.8	617.9	290.4	6.6	12.5	9.8	8.0	5196.7	188.6	192.8	0.3
CV	0.5	1.2	0.5	1.4	1.3	1.1	0.6	3.0	1.4	0.3	0.2	0.2
**1971–1990**												
Minimum	492	4	366	2.35	0.18	0.37	2.1	0.0005	11.2	54.6	107.0	1.1
Maximum	3810	576	3714	447	15.51	21.47	47.6	5.0	9898.1	808.6	1030.0	4.6
Mean	1794.9	102.3	1692.6	51.97	2.16	3.60	13.2	0.3	1121.8	438.1	726.5	1.8
Std. deviation	739.3	107.8	733.5	72.5	2.7	4.0	7.9	0.7	1611.1	176.6	202.4	0.7
CV	0.4	1.1	0.4	1.4	1.2	1.1	0.6	2.7	1.4	0.4	0.3	0.4

There were 49 and 82 recorded events in 1961–1969 and 1971–1990, respectively, included in the statistical analyses.

*CV* coefficient of variation; *T* flood event duration, in min; *T*_*p*_ time-to-peak, in min; *T*_*r*_ duration of recession, in min; *q*_*p*_ peak discharge, in m^3^ s^−1^; *H* runoff depth, in mm; *q*_*m*_ mean discharge, in m^3^ s^−1^; *FV* flow variability, which is defined as the ratio of the event-based flood peak discharge to mean discharge; *E* runoff erosion power, in m^4^ s^−1^; *SSY* area-specific sediment yield, in t km^−2^; *SCE* average suspended sediment concentration, in kg m^−3^; *MSCE* maximum suspended sediment concentration, in kg m^−3^; *SCV* sediment variability, which is defined as the ratio of the maximum to average suspended sediment concentration at the event timescale. The same note also applies to Table 2.

### Influence on intra-event-based flood regimes

#### Classification of flood events and the characteristics of baseline period flood regimes

**Figure 2:**
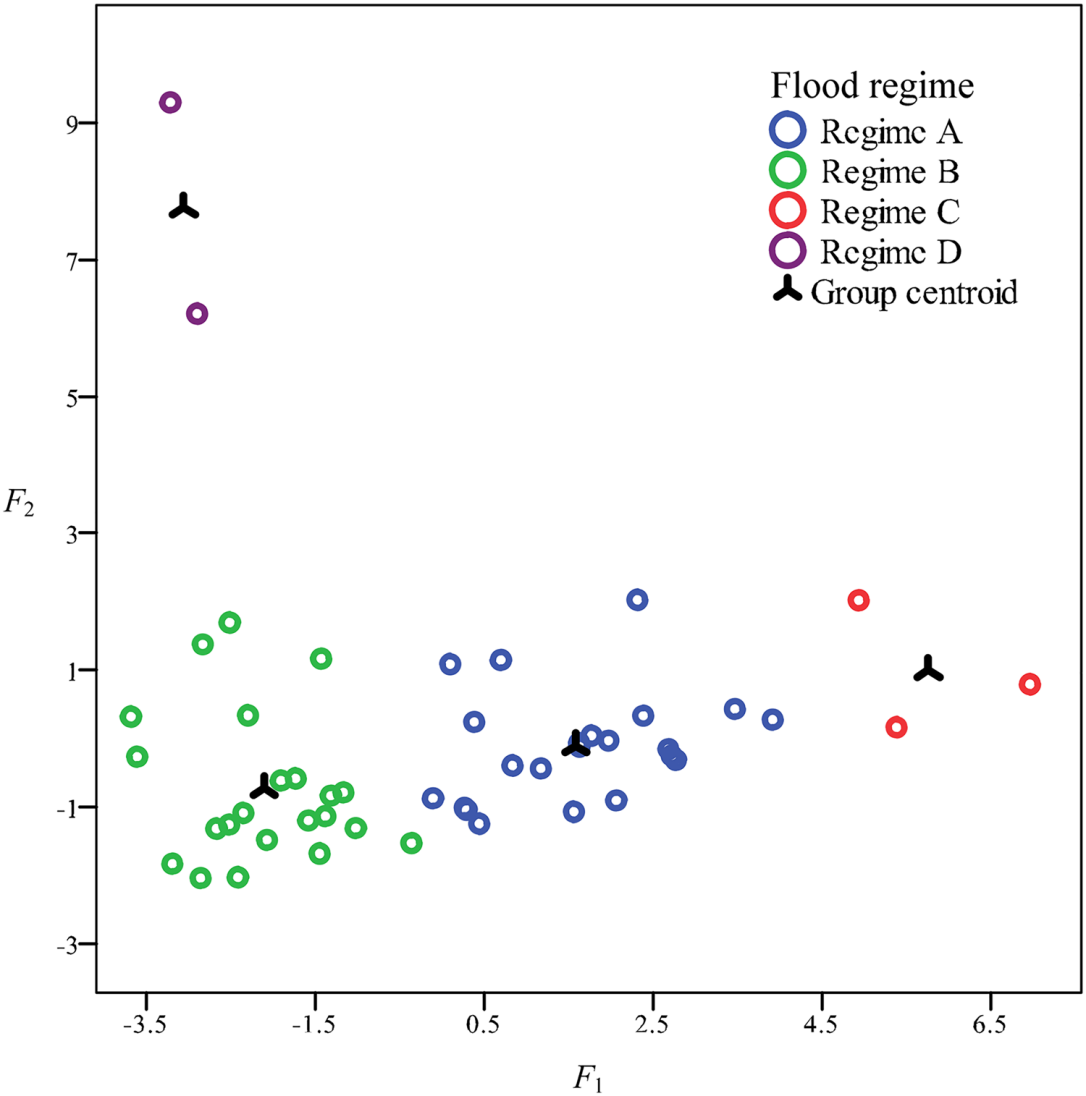
Discriminant analysis of different flood regimes in 1961–1969. *F*_*1*_ and *F*_*2*_ represent the scores of discriminant functions. Regime A: short flood duration and low erosive energy; Regime B: short flood duration and high erosive energy; Regime C: long flood duration and low erosive energy; Regime D: long flood duration and high erosive energy.

Figure 2 shows the clustering results of the flood events at the Caoping hydrological station in 1961–1969. The flood events were divided into 4 regimes with a significance level of *p* < 0.001. The data in the scatter diagrams of different discriminant functions were clustered, which indicated that the classification results were reasonable.

The discriminant functions were as follows:

$$F_{1} = 0.00{4}T + 0.00{1}q_{p} - 0.{22}H - {4}.{6}$$

$$F_{2} = 0.00{1}T - 0.00{1}q_{p} + 0.{292}H - {2}.{581}$$

$$F_{3} = 0.00{8}q_{p} - 0.{3}0{5}H - 0.{76}$$

The classification functions of the different regimes were as follows:

$$D_{1} = 0.0{25}T + 0.0{1}q_{p} - 0.{878}H - {23}.{927}$$

$$D_{2} = 0.0{11}T + 0.00{7}q_{p} - 0.{24}H - {6}.{495}$$

$$D_{3} = 0.0{4}T + 0.0{13}q_{p} - {1}.{456}H - {61}.{74}$$

$$D_{4} = 0.0{14}T + {2}.{445}H - {56}.{3}0{2}$$

where *F*_*1*_, *F*_*2*_, and *F*_*3*_ represent the scores of discriminant functions and *D*_*1*_, *D*_*2*_, *D*_*3*_ and *D*_*4*_ represent the classification scores of regimes A, B, C and D, respectively.

**Figure 3:**
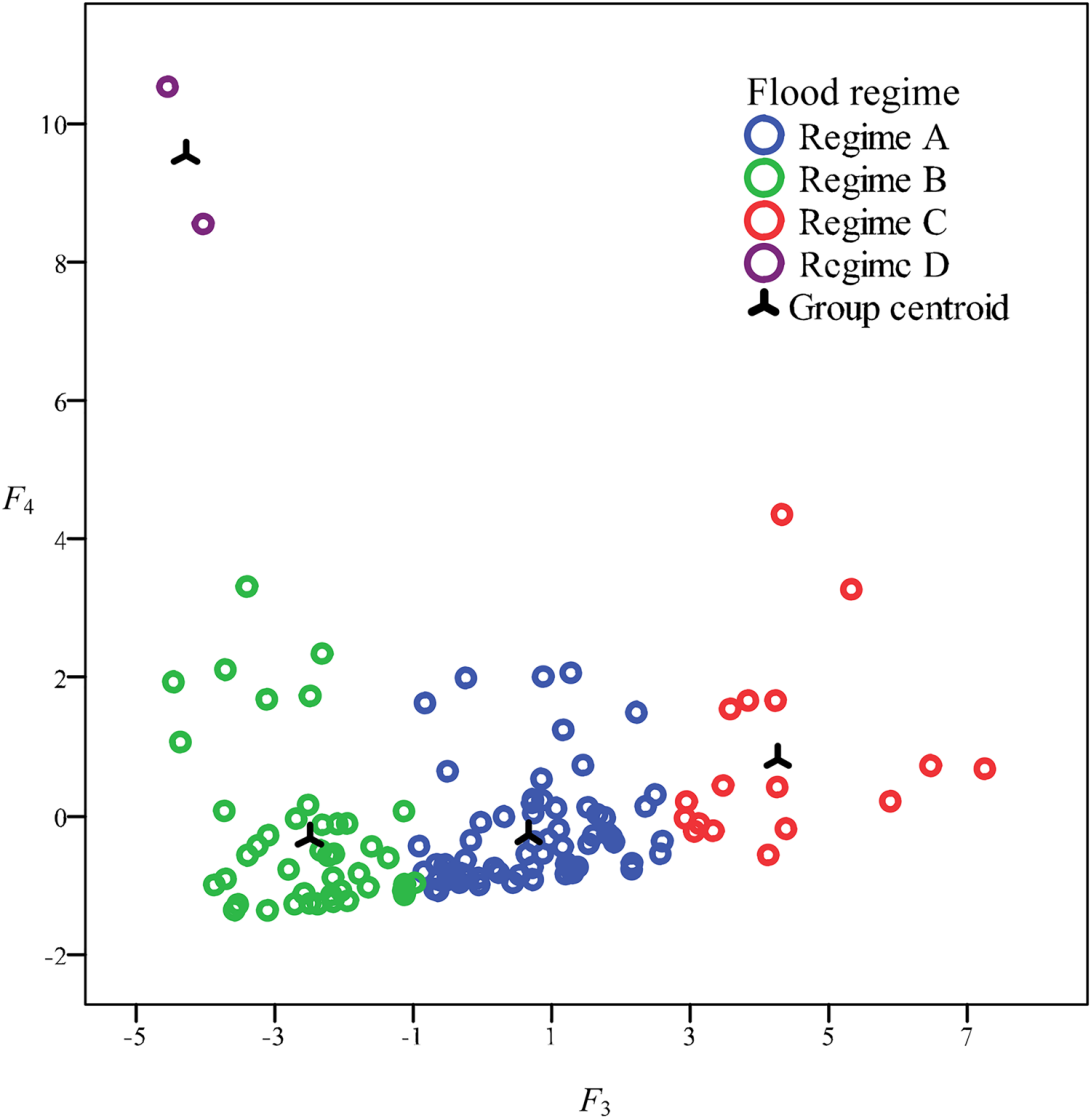
Discriminant analysis of different flood regimes in 1961–1990 (excluding 1971). *F*_*3*_ and *F*_*4*_ represent the scores of discriminant functions. Regime A: short flood duration and low erosive energy; Regime B: short flood duration and high erosive energy; Regime C: long flood duration and low erosive energy; Regime D: long flood duration and high erosive energy.

Based on the classification of the baseline period (1961–1969), the flood events of the measurement period (1971–1990) were discriminated with a significance level of *p* < 0.001; Fig. 3 presents the cluster results. The classification results were reasonable considering the scatter diagrams of the different discriminant functions.

The discriminant functions were as follows:

$$F_{4} = 0.00{3}T + 0.00{1}q_{p} - 0.{233}H - {5}.{2}$$

$$F_{5} = 0.00{1}q_{p} + 0.{288}H - {1}.{621}$$

$$F_{6} = 0.0{11}q_{p} - 0.{447}H - 0.{65}$$

The classification functions of the different regimes were as follows

$$D_{5} = 0.0{21}T + 0.0{14}q_{p} - {1}.{279}H - {19}.{749}$$

$$D_{6} = 0.0{1}T + 0.0{11}q_{p} - 0.{54}H - {6}.{1}$$

$$D_{7} = 0.0{34}T + 0.0{18}q_{p} - {1}.{779}H - {49}.{331}$$

$$D_{8} = 0.00{7}T + 0.0{18}q_{p} + {2}.{695}H - {64}.{322}$$

where *F*_*4*_, *F*_*5*_, and *F*_*6*_ represent the scores of discriminant functions and *D*_*5*_, *D*_*6*_, *D*_*7*_ and *D*_*8*_ represent the classification scores of regimes A, B, C and D, respectively.

Table 2 describes the classification results and the characteristics of different flood regimes. During the baseline period, the flood durations of regimes A and B were short, whereas the flood durations of regimes C and D were long. The *q*_*p*_, *H*, *E*, *SSY* and *SCE* of regime A, which accounted for 42.86% of all flood events, were small. The *T* of regime B, which accounted for 44.90% of the flood events, was the shortest, but the *q*_*p*_, *H*, *E*, *SSY* and *SCE* of regime B were large. Regime C, which accounted for 8.16% of all flood events, had the longest *T*, but the *q*_*p*_, *H*, *E*, *SSY* and *SCE* of regime C were small. The *q*_*p*_, *H*, *E*, *SSY* and *SCE* of regime D, which represented 4.08% of all flood events, were the largest. The runoff erosive energies of regimes A and C were smaller than those of regimes B and D, respectively.

**Table 2:** Descriptive statistics of the characteristics of event-based flood flows and sediment under different flood regimes in 1961–1990 (excluding 1970).

Year	Regime/*N*	*T*	*T*_*p*_	*T*_*r*_	*q*_*p*_	*H*	*q*_*m*_	*FV*	*E*	*SSY*	*SCE*	*MSCE*	*SCV*	PR
1959–1969	A/21	1878.81	293.48	1585.33	129.00	3.40	5.52	21.50	0.68	2388.62	643.74	823.29	1.33	42.86
	B/22	906.59	129.18	777.41	215.20	4.26	14.41	12.39	1.72	3229.82	661.17	810.25	1.27	44.90
	C/4	2963.25	423.75	2539.50	77.45	3.43	3.60	23.30	0.33	1731.95	553.70	848.00	1.58	8.16
	D/2	2105.50	286.00	1819.50	1256.50	32.33	47.88	26.22	39.58	25,108.29	776.84	961.00	1.24	4.08
1971–1990	A/47	1822.68	100.23	1722.45	38.42	1.69	2.66	12.77	0.14	801.30	414.27	740.87	1.97	57.32
	B/22	994.91	93.64	901.27	49.04	1.64	5.02	9.15	0.22	907.39	446.32	655.86	1.62	26.83
	C/13	3048.31	124.46	2923.85	105.92	4.78	4.56	21.43	0.87	2643.32	510.20	793.92	1.78	15.85

Regime A: short flood duration and low erosive energy; Regime B: short flood duration and high erosive energy; Regime C: long flood duration and low erosive energy; Regime D: long flood duration and high erosive energy. *N*, the number of recorded flood events in the regime; PR, the proportion of the number of recorded flood regimes to the total flood number at different times, in %. Other indicators are as defined in Table 1.

#### Effect on intra-event-based flood regimes

The average *T* of the measurement period was 1.17 times longer than the *T* of the baseline period. In addition, *q*_*p*_ decreased by 75.2% in the measurement period. *E* in the measurement period accounted for only 10.2% of that in the baseline period (Table 1). Consequently, in the measurement period, the flood events transitioned from regimes B and D, which have high erosive energy, to regimes A and C, which have low erosive energy. Compared with those in the baseline period, the proportions of regime A and regime C flood events increased by 33.7% and 94.2%, respectively, during the measurement period; regime B flood events decreased from 44.9% to 26.8%, and regime D flood events did not occur in the measurement period.

Because the conservation measures weakened the erosive energy of runoff, other characteristics within the same regime changed between the measurement period and baseline period. The *q*_*p*_, *H*, *SSY* and *SCE* of regimes A and B were smaller in the measurement period than in the baseline period, and the *E* of regimes A and B decreased by 79.6% and 87.4%, respectively, in the measurement period. Due to the increase in *T* and the decrease in erosion in the measurement period, regime D, which is the regime with the maximum erosive ability, transitioned into regime C, which has a long *T* and low erosive energy. Therefore, the variables of regime C, such as *T*, *q*_*p*_, *H*, *q*_*m*_, *SSY* and *SCE*, increased in the measurement period compared with the baseline period. In addition, the *q*_*p*_, *H*, *q*_*m*_, *SSY* and *SCE* of regime C were larger than those of regimes A and B in the measurement period and smaller than those of regime D and regimes C/D in the baseline period.

## Discussion

### Effect on sediment yield

Comparisons of the runoff and sediment characteristics between the baseline period (1961–1969) and measurement period (1971–1990) showed that *SSY*, *SCE* and *MSCE* decreased by 69.2%, 33.3% and 11.9%, respectively, in the measurement period. The extensive implementation of afforestation, grass planting and terracing measures and, especially, the large-scale construction of check dams^16,39,40^ has profoundly affected the physical characteristics of the underlying surface, the erosional environment and the surface hydrological processes of the watershed, thus changing the total amount and temporal distribution of flood runoff. Accordingly, these measures have resulted in the redistribution of runoff erosion energy and changed the dynamic processes to control soil and water loss.

The conservation measures delayed runoff formation, increased soil infiltration and intercepted rain^20,41^, thereby causing *q*_*p*_, *H* and *q*_*m*_ to decrease by 75.2%, 56.0% and 68.0%, respectively. Hence, the runoff erosion energy was reduced; for example, *E* in the measurement period was only 10.2% of that in the baseline period.

After repeated trial and error, selection of the *T*, *q*_*p*_, *H* indicators and cluster and discriminant analyses, the flood events in the baseline period (1961–1969) at the Caoping hydrological station were classified into four regimes, and the runoff and sediment characteristics in the different regimes were investigated. The flood durations of regimes A and B were shorter, whereas those of regimes C and D were longer. In addition, regimes A and C produced less sediment yield and lower soil erosion energy; in contrast, regimes B and D produced more sediment yield and higher soil erosion energy. This research illustrated that different sediment characteristics occurred in different flood regimes. Therefore, the conservation measures achieved the purpose of reducing sediment yield by not only reducing runoff amount or soil erosion energy but also transforming flood regimes, for example, transforming a high-sediment-yield regime into low-sediment-yield regime.

### Effect on intra-event-based flood regimes

The conservation measures prolonged the flood duration, decreased the peak discharge and runoff depth, and transformed the high-sediment-yield regimes B and D into the low-sediment-yield regimes A and C; notably, regime D, which had the most sediment yield, did not occur in the measurement period. Because regime D did not occur in this period and because the conservation measures transformed regime D into regime C, regimes C and D in the baseline period were merged. By nonlinear fitting of *SSY* and *E* in the baseline and measurement periods, a power function relationship between *SSY* and *E*, with all *R*^2^ > 0.8, was discovered. The *SSY*-*E* regression lines were compared between the baseline and measurement periods in different regimes. The *SSY* and *E* relationship in regime A obviously changed in the measurement period; however, there was no obvious change in regime B or C/D. This result indicated that conservation measures could change the runoff and sediment relationship in regimes with low sediment yields. However, because the relationship between runoff and sediment remains approximately constant and because the “self-regulation” of flood runoff shows limited potential for sediment reduction^24^, the conservation measures in regimes with high sediment yields could reduce sediment yield only by reducing the runoff amount or soil erosion energy. Moreover, the conservation measures weakened the runoff erosion energy; for example, the concentration range of *E* (75% of the number of flood events) in the measurement period was less than that of the baseline period in all regimes.

### Mechanism analysis based on runoff erosion power

*H* and *q*_*p*_ are two important parameters that reflect intra-event flood characteristics. *H* represents the total amount of runoff generated by heavy rain in the basin, which indirectly reflects the precipitation amount and the influence of the underlying surface of the watershed on the redistribution of rainfall. *q*_*p*_ represents the flood intensity, which indirectly reflects the temporal and spatial distribution of rainfall and the effect of the underlying surface of the watershed on the confluence process. Therefore, *E*, which is the product of runoff depth and flood peak flow^28^, was chosen to represent erosion energy to explore the relationship between area-specific sediment yield (SSY) and *E* under different regimes in the baseline period (1961–1969) and the measurement period (1971–1990).

**Figure 4:**
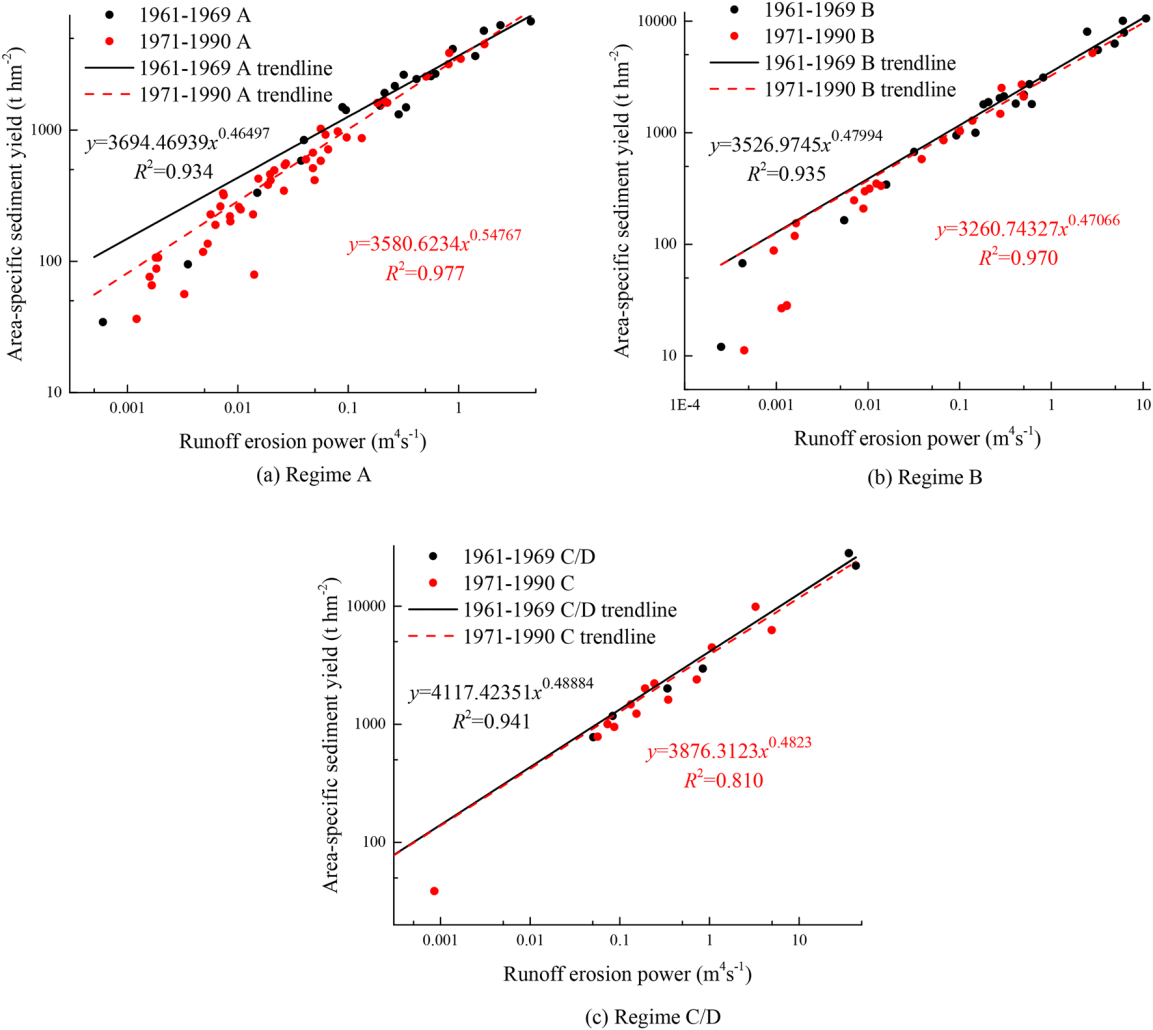
Correlation of area-specific sediment yield and runoff erosion power of different event-based flood regimes at different times. Regime A: short flood duration and low erosive energy; Regime B: short flood duration and high erosive energy; Regime C: long flood duration and low erosive energy; Regime D: long flood duration and high erosive energy.

Because conservation measures prolong flood duration and reduce runoff erosion energy, regime D transitioned to regime C in the measurement period. In addition, given that the number of events in regimes C and D in the baseline period was low, regimes C and D in the baseline period were combined for the regression analysis of the *SSY*-*H* relationship. As shown in Fig. 4, the regression line for the baseline period, during which no large-scale conservation measures were carried out, plotted higher than that for the measurement period, indicating that the conservation measures generally reduced sediment yield in the watershed. Additionally, in regime A, the *E* of 75% of the flood events in the baseline period ranged from 0.095 to 4.501 m^4^ s^−1^, whereas that in the measurement period was in the range of 0.001–0.062 m^4^ s^−1^. In addition, the regression lines were obviously different between the baseline and measurement periods. The *SSY* of the measurement period was less than that of the baseline period with the same *E*, although the difference gradually decreased as *E* increased. In other words, the conservation measures not only reduced the runoff erosion energy but also changed the relationship between *SSY* and *E*. In the plots for regimes B and C/D, the regression lines of the baseline and measurement periods were almost coincident; however, those of the measurement period plotted slightly lower than those of the baseline period, indicating that in regimes B and C/D, the reductions in soil and water loss were not driven by changes in the relationship of *SSY* and *E*. In the baseline period, the *E* values of most flood events (75%) were 0.149–10.765 m^4^ s^−1^ and 0.341–43.153 m^4^ s^−1^ in regime B and regime C/D, respectively. However, in the measurement period, the *E* of most flood events (75%) varied from 0.001 to 0.101 m^4^ s^−1^ in regime B and varied from 0.001 to 0.347 m^4^ s^−1^ in regime C. Hence, the conservation measures reduced the sediment yield mainly by reducing the runoff erosion energy.

## Conclusion

In total, 49 flood events from 1961 to 1969 (baseline period) and 82 flood events from 1971 to 1990 (measurement period) were selected for assessment. Compared with those in the baseline period, the runoff characteristics (*q*_*p*_, *H*, *q*_*m*_ and *E*) and sediment characteristics (*SSY*, *SCE* and *MSCE*) of floods in all regimes benefited from the conservation measures in the measurement period.

Based on hierarchical cluster analysis and discriminant analysis, the flood events of the Caoping hydrological station were classified into four regimes: regime A, with short flood duration and low erosive energy; regime B, with short flood duration and high erosive energy; regime C, with long flood duration and low erosive energy; and regime D, with long flood duration and high erosive energy.

The conservation measures transformed the high-sediment-yield B and D regimes to the low-sediment-yield A and C regimes and changed the relationship between *SSY* and *E* in regime A. The conservation measures had little effect on the *SSY*-*E* relationship in the high-sediment-yield B and C/D regimes. Additionally, *E* was lower in the measurement period than in the baseline period in all regimes. This study provides evidence of the mechanism of runoff regulation and the sediment yield reduction benefit of conservation measures.
